# Suppression of protein degradation by leucine requires its conversion to β-hydroxy-β-methyl butyrate in C2C12 myotubes

**DOI:** 10.18632/aging.102509

**Published:** 2019-12-24

**Authors:** Yehui Duan, Yinzhao Zhong, Bo Song, Changbing Zheng, Kang Xu, Xiangfeng Kong, Fengna Li

**Affiliations:** 1 Hunan Provincial Key Laboratory of Animal Nutritional Physiology and Metabolic Process, Key Laboratory of Agro-ecological Processes in Subtropical Region, Institute of Subtropical Agriculture, Chinese Academy of Sciences, Hunan Provincial Engineering Research Center for Healthy Livestock and Poultry Production, Scientific Observing and Experimental Station of Animal Nutrition and Feed Science in South-Central, Ministry of Agriculture, National Engineering Laboratory for Pollution Control and Waste Utilization in Livestock and Poultry Production, Changsha, Hunan, P. R. China; 2 Guangdong Provincial Key Laboratory of Animal Nutrition Regulation, South China Agricultural University, Guangzhou, Guangdong, China; 3 Hunan Co-Innovation Center of Animal Production Safety, CICAPS, Hunan Collaborative Innovation Center for Utilization of Botanical Functional Ingredients, Changsha, Hunan, China

**Keywords:** β-hydroxy-β-methyl butyrate, protein degradation, mitochondrial function, leucine, C2C12 myotubes

## Abstract

The aims of this study were to investigate whether the inhibitory effect of Leucine (Leu) on starvation-induced protein degradation was mediated by its metabolite β-hydroxy-β-methyl butyrate (HMB), and to explore the mechanisms involved. The results showed that the beneficial effects of Leu on protein degradation and the oxygen consumption rate (OCR) of cells were observed at low levels (0.5 mM) rather than at high levels (10 mM). However, these effects were inferior to those of HMB. Moreover, HMB was able to increase/decrease the proportion of MyHC I/MyHC IIb protein expression, respectively. In these KICD-transfected cells, Leu was approximately as effective as HMB in inhibiting protein degradation and increasing the OCR as well as MyHC I protein expression of cells, and these effects of Leu were reverted to a normal state by mesotrione, a specific suppressor of KICD. In conclusion, HMB seems to be an active metabolite of Leu to suppress muscle protein degradation in a starvation model, and the mechanisms may be associated with improved mitochondrial oxidative capacity in muscle cells.

## INTRODUCTION

Skeletal muscle comprises approximately 40% of total body weight and contributes significantly to multiple bodily functions [1]. Since energy in the form of adenosine triphosphate (ATP) is required for all muscle actions, skeletal muscle’s efficient functioning is directly associated with its intact energy metabolism [2]. Mitochondria are the powerhouse of the cell and their main function is ATP production. Dysregulation in mitochondrial oxidative phosphorylation is closely related to muscle atrophic diseases, including the muscular dystrophies, sarcopenia, and cachexia [3–5]. Skeletal muscle that accompanies these diseases is manifested by the loss of skeletal muscle oxidative capacity, defined by the ability to oxidize nutrients to obtain energy [6]. Conversely, improving mitochondrial function brings beneficial effects on muscle oxidative capacity in disease states, thus contributing to improved muscle health and whole-body health [7]. As such, the characterization of compounds that can improve mitochondrial function and increase the oxidative capability of muscle fibers could provide the foundation for the development of therapeutic nutraceuticals that attenuate muscle atrophy.

Leucine (Leu), a branched-chain amino acid (BCAA), has been viewed as a regulator of mitochondrial function and oxidative capacity of muscle fibers [8, 9]. Evidence for this is provided by observations that mitochondrial density and oxidative capacity of C2C12 myoblasts are enhanced in response to Leu treatment (0.1-0.5 mM) [10]. Similar results are also obtained in β-hydroxy-β-methyl-butyrate (HMB, a Leu metabolite)-treated C2C12 myotubes and older adults [11–13]. More interestingly, we recently found that HMB (50 μM) is more superior than Leu (0.5 mM) in effectively improving mitochondrial function of C2C12 myotubes [12]. However, these *in vitro* studies were mainly performed under anabolic conditions. No study has systematically compared the effects of Leu and its metabolites on mitochondrial function under catabolic conditions. Moreover, it remains unclear whether improved mitochondrial function stimulated by Leu and its metabolites is accompanied by increased oxidative capability of muscle fibers and muscle health. Therefore, further investigation is certainly warranted.

Interestingly, both Leu and its metabolites (α-ketoisocaproate (KIC) and HMB) are capable of ameliorating protein degradation in skeletal muscle [14–18]. Furthermore, our recent studies demonstrate for the first time that the inhibitory effects of HMB (50 μM) on protein degradation is more potent than Leu (0.5 mM, a concentration within a range that is physiologically relevant) [19]. The mechanism of HMB action is associated with PI3K/Akt signaling pathway [19–21]. Intrigued by these interesting observations, we asked whether the protective effect of HMB is still more effective than that of Leu when its treatment concentration is far beyond physiological limits, and whether HMB mediates the inhibitory effect of Leu on protein degradation.

Therefore, in the present study, we investigated the effects of Leu (within or above a range that is physiologically relevant) versus KIC and HMB on protein degradation and mitochondrial function in C2C12 myotubes under catabolic conditions. Our results showed that the regulatory effects of HMB on protein degradation and mitochondrial function are more potent than those of Leu (within a range that is physiologically relevant, but not above this range). Then, to determine whether HMB mediates these effects of Leu, we over-expressed the enzyme α-keto isocaproate dioxygenase (KICD, a key enzyme required for the conversion of Leu to HMB) in C2C12 cells. Effectively, Leu potentiated its effects on protein degradation and mitochondrial function in these transfected cells. Taken together, our results seem to suggest that Leu effects on muscle protein degradation and mitochondrial function are in fact mediated by the metabolite HMB under our experimental setting.

## RESULTS

### HMB was superior to Leu and KIC in effectively ameliorating starvation-induced muscle protein degradation in C2C12 myotubes

**Figure 1:**
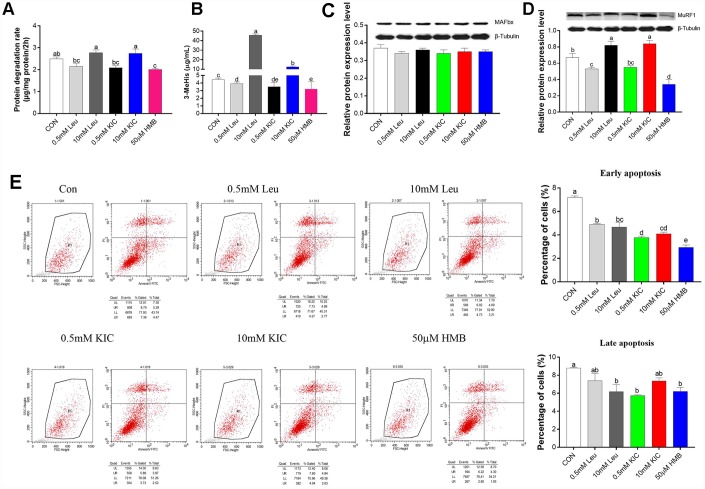
Effects of Leu (0.5 mM or 10 mM), KIC (0.5 mM or 10 mM), and HMB (50 μM) on (**A**) protein degradation, (**B**) media 3-MeHis, (**C**) MAFbx protein expression, (**D**) MuRF1 protein expression, and (**E**) cell apoptosis in C2C12 myotubes. Results are expressed as mean ± SEM. Different letters (**a**, **b**, **c**) indicated significant differences (*P* < 0.05). CON, control; HMB, β-hydroxy-β-methyl butyrate; KIC, α-ketoisocaproate; Leu, leucine.

As shown in Figure 1A–1D, treatment with Leu or KIC at the concentration of 10 mM increased the protein degradation rate (11.24% and 10.04%, respectively), the protein expression of MuRF1 (22.39% and 25.37%, respectively), and the 3-MeHis concentration (10.21-fold and 2.79-fold, respectively) (*P* < 0.05) in comparison with the control group. When used at a concentration of 0.5 mM, Leu or KIC induced a reduction in the protein degradation rate (14.06% and 16.06%, respectively), the protein expression of MuRF1 (20.90% and 17.91%, respectively), and in the 3-MeHis concentration (12.30% and 21.02%, respectively) (*P* < 0.05) compared to control group. The protein degradation rate, the protein expression of MuRF1, and 3-MeHis concentration were significantly decreased (*P* < 0.05 for all) by 19.27%, 49.25% and 27.96%, respectively, after HMB treatment. Additionally, we observed significant decreases in the percentages of both early and late apoptotic cells with 50 μM of HMB treatment compared to control group (Figure 1C, *P* < 0.05). Therefore, among Leu, KIC, and HMB, HMB inhibited protein degradation to the greatest extent.

### Leu, KIC, and HMB differently affected mRNA and protein expression of skeletal muscle fiber type-related genes

**Figure 2:**
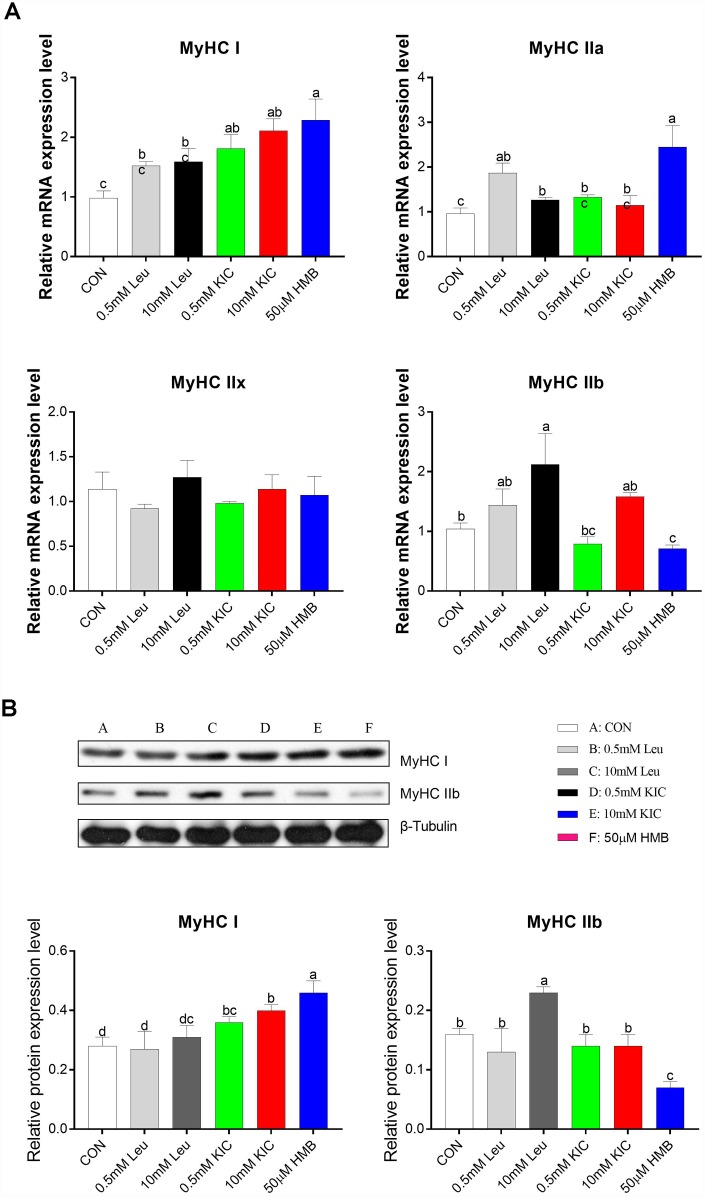
Effects of Leu (0.5 mM or 10 mM), KIC (0.5 mM or 10 mM), and HMB (50 μM) on the gene expression of myosin heavy chain isoform (MyHC I, IIa, IIx, and IIb) (**A**) and the protein expression of MyHCI and MyHC IIb (**B**). Results are expressed as mean ± SEM. Different letters (**a**, **b**, **c**) indicated significant differences (*P* < 0.05). CON, control; HMB, β-hydroxy-β-methyl butyrate; KIC, α-ketoisocaproate; Leu, leucine.

In this starvation model, to explore the effects of Leu and its metabolites on muscle fiber characteristics, we analyzed the relative mRNA abundance and protein expression of myosin heavy chain isoform (MyHC I, IIa, IIx, and IIb) in C2C12 myotubes. Compared to the control group, HMB upregulated the mRNA abundance of slow-twitch fiber-related genes MyHC I and MyHC IIa (2.34-fold and 2.55-fold, respectively; *P* < 0.05), whereas it downregulated the mRNA abundance of the fast-twitch fiber-related gene MyHC IIb (31.73%, *P* < 0.05, Figure 2A). Consistently, we found that HMB elevated MyHC I protein expression and reduced MyHC IIb protein expression (*P* < 0.05, Figure 2B). These results suggest that HMB induces a fast-twitch to slow-twitch transition in myotubes. Furthermore, the 10 mM Leu group significantly increased the gene and protein expression of MyHC IIb relative to the control group (*P* < 0.05, Figure 2).

### Leu and HMB effects on mitochondrial function of C2C12 myotubes

**Figure 3:**
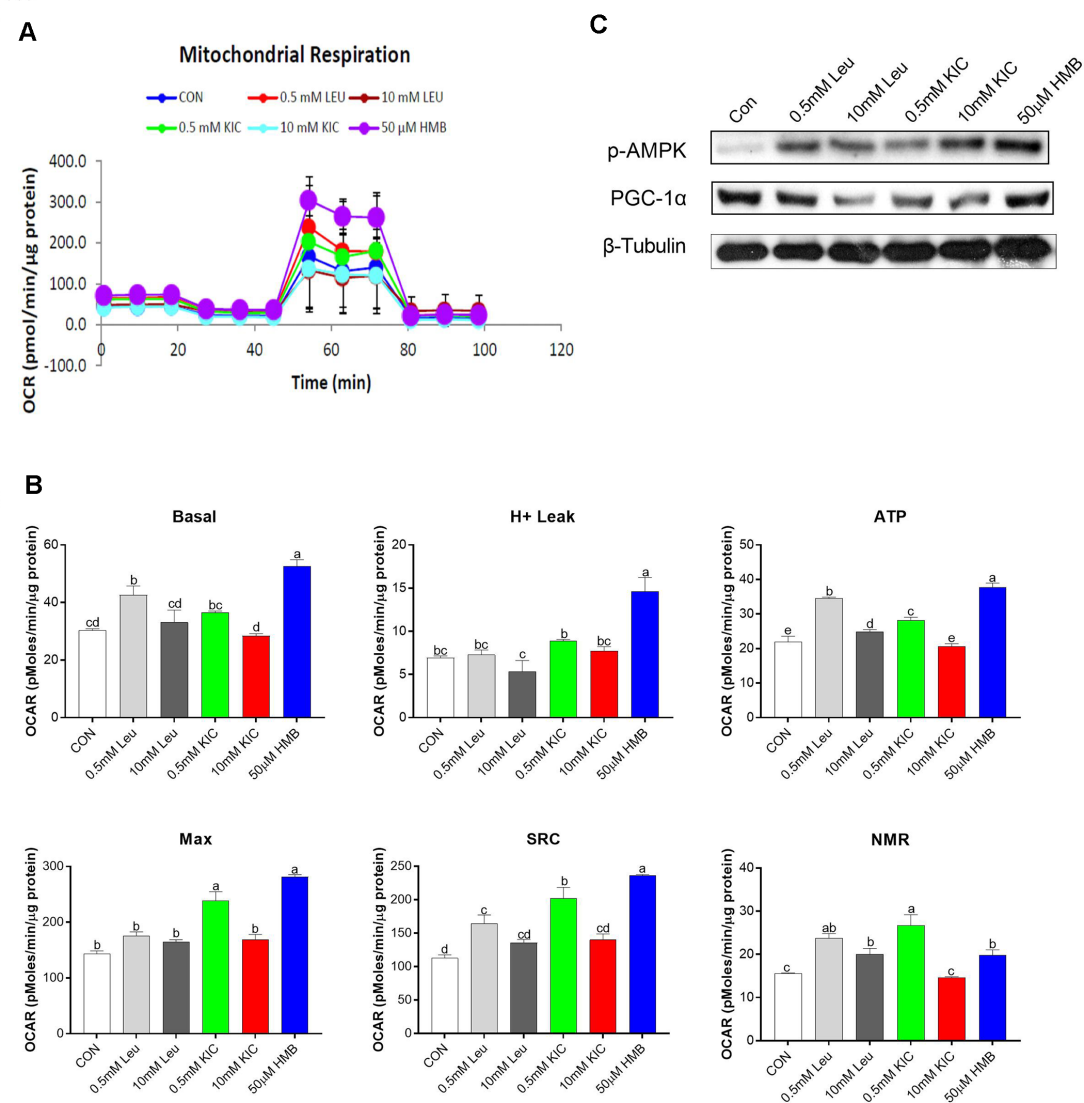
Effects of Leu (0.5 mM or 10 mM), KIC (0.5 mM or 10 mM), and HMB (50 μM) on (**A**–**B**) oxygen consumption rate (OCR) of C2C12 myotubes and (**C**) protein expression of AMPKα and PGC-α. (**A**) Represents mitochondrial OCR curves obtained from different conditions. (**B**) Basal, basal respiration; H^+^ leak; ATP, ATP production; Max, maximum respiration; SRC, spare respiration capacity; and NMR, non-mitochondrial respiration of C2C12 myotubes under different treatments, respectively. Results are expressed as mean ± SEM. Different letters (**a**, **b**, **c**) indicated significant differences (*P* < 0.05). CON, control; HMB, β-hydroxy-β-methyl butyrate; KIC, α-ketoisocaproate; Leu, leucine.

In this starvation model, to determine the effect of Leu and its metabolites on mitochondrial respiration, OCR was analyzed using a SeaHorse XF analyzer in C2C12 myotubes. In this study, the greatest increase in the OCR of cells occurred in the HMB group, followed by 0.5 mM Leu and KIC (Figure 3A). In detail, as shown in Figure 3B, in comparison with the control group, Leu (0.5 mM) and HMB treatment increased basal mitochondrial respiration by 1.40-fold and 1.74-fold (*P* < 0.05), enhanced ATP production by 1.57-fold and 1.71-fold (*P* < 0.05), augmented the spare respiration capacity (SRC) by 1.46-fold and 2.09-fold (*P* < 0.05), and elevated the NMR by 1.52-fold and 1.27-fold (*P* < 0.05), respectively. These parameters were not significantly different between the control group and other groups (*P* > 0.05). Additionally, the H^+^ leak and Max of HMB-treated myotubes were elevated by 2.11-fold and 1.97-fold (*P* < 0.05), respectively, compared to the control group, whereas Leu treatment (0.5 mM or 10 mM) failed to exert any effects (*P* > 0.05). Furthermore, we measured the protein expression of several regulators of mitochondrial biogenesis. As revealed in Figure 3C, HMB treatment induced obvious increases in the protein expression of p-AMPK and PGC-1α.

### Protein degradation and mitochondrial function in C2C12 myotubes over-expressing KICD

**Figure 4:**
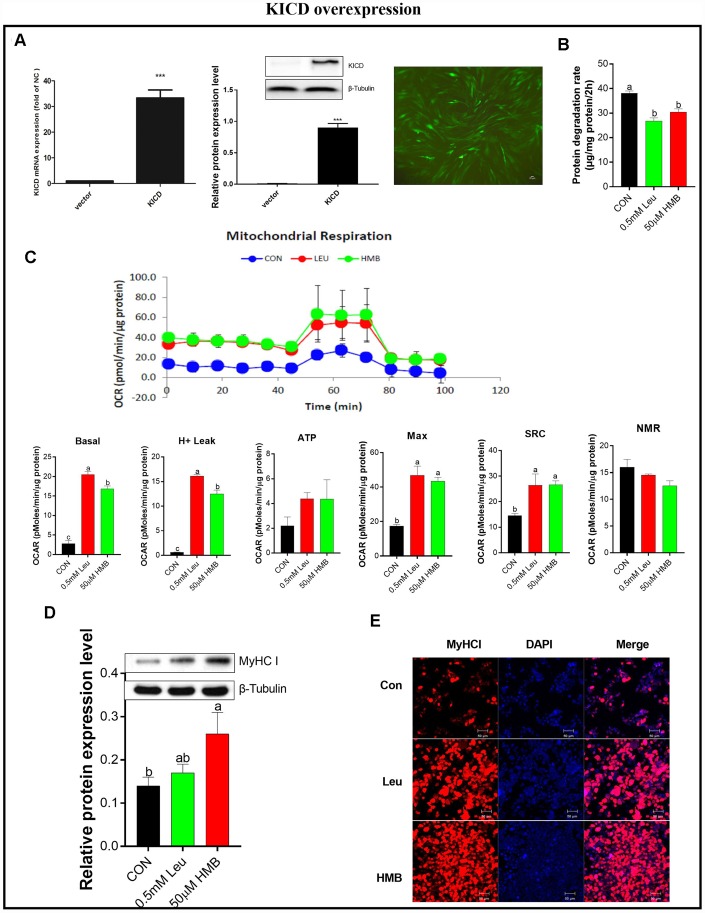
**Effects of Leu and HMB on protein degradation, the oxygen consumption rate (OCR), and MyHC I protein expression and immunofluorescence intensity in KICD-transfected C2C12 myotubes.** (**A**) RT-PCR and Western blot analysis of KICD from non-transfected and transfected C2C12 myotubes is shown. (**B**) Protein degradation rates in the presence of 0.5 mM Leu and 50 μM HMB. (**C**) The OCR of cells in the presence of 0.5 mM Leu and 50 μM HMB. (**D**) MyHC I protein expression in the presence of 0.5 mM Leu and 50 μM HMB. (**E**) MyHC I immunofluorescence intensity in the presence of 0.5 mM Leu and 50 μM HMB. Results are expressed as mean ± SEM. Different letters (**a**, **b**, **c**) indicated significant differences (*P* < 0.05). CON, control; HMB, β-hydroxy-β-methyl butyrate; KIC, α-ketoisocaproate; Leu, leucine.

Since KICD is mainly expressed in the liver and kidney, the conversion of Leu to HMB is limited in muscle and mainly occurs in the liver [24, 32]. Therefore under our *in vitro* experimental conditions, we hypothesized that the regulatory effects of Leu on protein degradation and mitochondrial function would be due to the limited rate of Leu metabolism to HMB in muscle, suggesting HMB as an active Leu metabolite. To explore the effects of a higher rate of conversion of Leu to HMB, we transfected C2C12 cells with an expression plasmid, termed pKICD coding for the rat enzyme, and the KICD expression was confirmed by RT-PCR and western blot (Figure 4A). The KICD levels were significantly higher in pKICD C2C12 transfected cells than in untransfected cells.

The effects of Leu or HMB treatment on protein degradation and mitochondrial function were assayed on C2C12 cells over-expressing KICD under non-amino-acid deprivation. In these transfected cells, HMB retains the capability to inhibit protein degradation and to improve mitochondrial function. Interestingly, treatment with 0.5 mM Leu induced a significant reduction in protein degradation and a marked increase in mitochondrial function (*P* < 0.05, Figure 4B–4C). This effect is prominent given that Leu addition to C2C12 non-transfected myotubes induced a marked lower effect in comparison with HMB in protein degradation and mitochondrial function (Figures 1 and 3).

Following this, we measured the effects of Leu or HMB treatment on MyHC I expression in the pKICD transfected cells (Figure 4D–4E). In these cells, incubation of the cells with Leu increased the protein expression and the fluorescent density of MyHC I at levels similar to those in HMB-treated cells.

**Figure 5:**
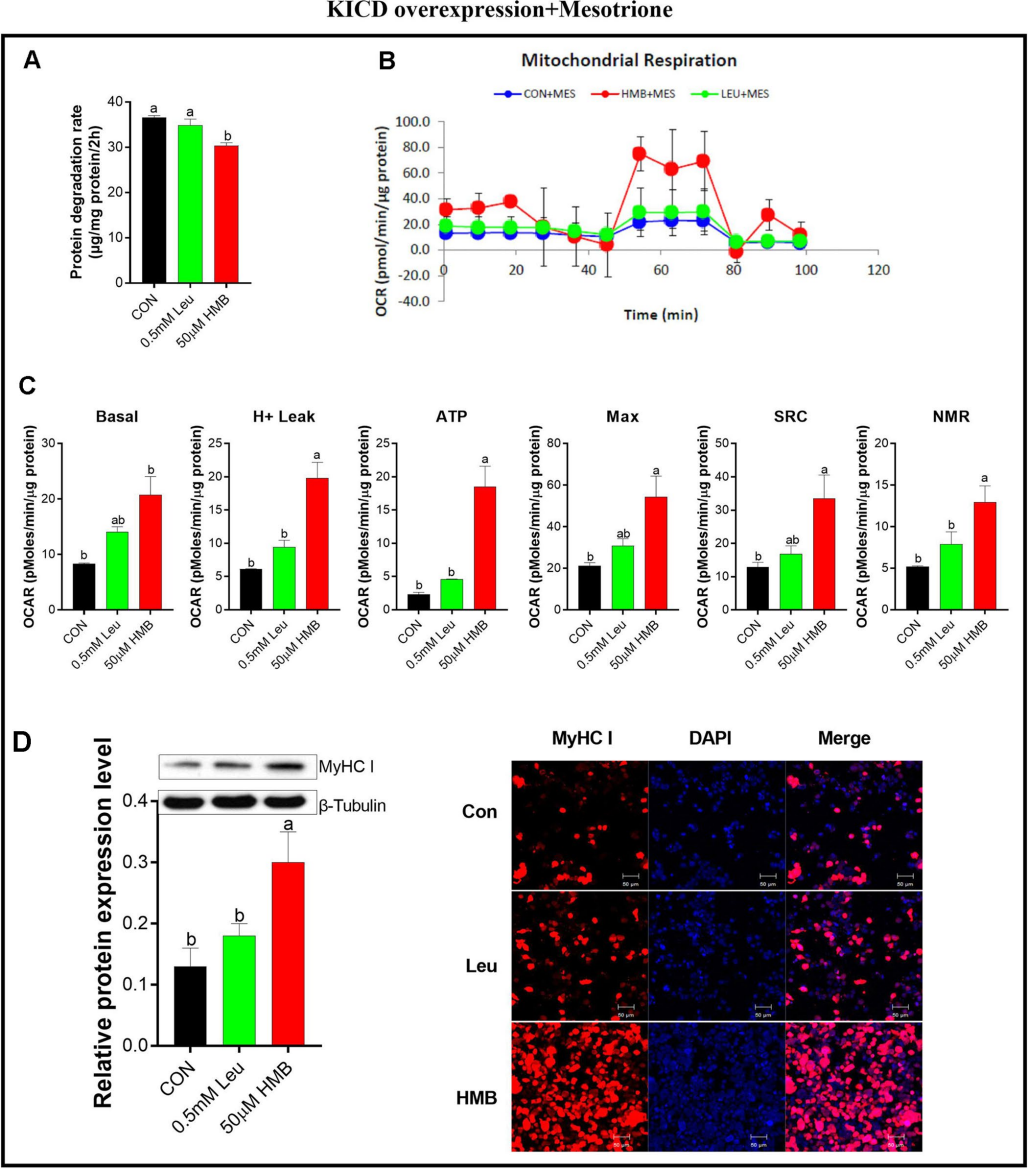
**Effect s of Leu and HMB on protein degradation, the oxygen consumption rate (OCR), and MyHC I protein expression and immunofluorescence intensity in KICD-transfected C2C12 myotubes in the presence of 1 μM mesotrione.** (**A**) Protein degradation rates in the presence of 0.5 mM Leu and 50 μM HMB. (**B**–**C**) The OCR of cells in the presence of 0.5 mM Leu and 50 μM HMB. (**C**–**D**) MyHC I protein expression and immunofluorescence intensity in the presence of 0.5 mM Leu and 50 μM HMB. Results are expressed as mean ± SEM. Different letters (**a**, **b**, **c**) indicated significant differences (*P* < 0.05). CON, control; HMB, β-hydroxy-β-methyl butyrate; KIC, α-ketoisocaproate; Leu, leucine.

To confirm the importance of KICD on Leu-induced effects, protein degradation was assayed in the presence of 1 μM mesotrione (a specific KICD inhibitor) [33] in the C2C12-transfected cells (Figure 5). Pre-incubation with mesotrione blocked the reduction in protein degradation due to Leu, whereas it has no significant effects on HMB-treated cells (Figure 5A). Meanwhile, pre-incubation with mesotrione significantly reduced the effects of Leu on the OCR of cells to the levels observed in the untransfected cells (Figure 5B and 5C). In the presence of mesotrione, HMB retained the capability to enhance the OCR of these transfected cells in comparison with the control group (*P* < 0.05). Alterations in the protein expression and the fluorescent density of MyHC I showed the same trends as those of mitochondrial respiratory function.

## DISCUSSION

Muscle atrophy occurs mainly due to a larger increase in protein degradation. And intriguingly, the present study provides evidence that treatment with low concentrations of Leu or KIC (0.5 mM) inhibited starvation-induced protein degradation in C2C12 myotubes, while further increasing Leu or KIC concentrations to 10 mM failed to elicit an additional response. Although the reason for this observation is not clear, it is possible that an excess supply of Leu results in an imbalance of BCAAs, which may further promote protein degradation and impair muscle growth. Because the same enzymes are required for BCAAs in their catabolic pathways, and the excess supply of one BCAA may affect the requirements of the other BCAAs [34]. These results suggest that Leu or KIC can be regarded as possible adjuncts in nutritional programs to ameliorate protein degradation, but Leu or KIC may be ineffective at very high doses. Our current results fit well with a recent study reporting that Leu or KIC at concentrations of 0.5 mM is capable of repressing proteolysis, although their inhibitory effects are inferior to those of HMB [19]. However, contrary to what was observed in the present study, previous studies pointed out that the concentrations of at least 5 mM were required for Leu to increase protein synthesis [35]. Therefore, our understanding of Leu effects on protein metabolism was further advanced with the identification of its high levels not as a potent regulator for protein degradation under catabolic conditions, and we employed the Leu concentration of 0.5 mM in the following experiments.

Moreover, several previous studies report that unlike in healthy states, Leu has little ability to reduce the protein degradation rate of muscle cells under catabolic states [36, 37]. Consistently, the current results showed that the inhibitory effects of Leu were weak and inferior to those of HMB. Unlike Leu, HMB is potent repressor of proteolysis in catabolic states [26, 38, 39]. Total protein degradation induced by tumor necrosis factor-α and angiotensin II was completely attenuated by HMB (50 μM) [25]. Based on these observations, we hypothesized that in catabolic states, the transamination of Leu to HMB in muscle cells is the limiting step to obtain a complete response on protein degradation. To test this possibility, we transfected C2C12 myotubes with a plasmid pKICD to over-express this key enzyme for the conversion of Leu to HMB, since KICD is mainly expressed in the liver [23]. In addition, the use of this muscle cell line would allow us to differentiate between systemic and muscle specific Leu and HMB effects.

Over-expressing KICD in C2C12 myotubes exerted a key role in the potentiation of Leu effects since in these transfected cells, Leu decreased protein degradation to the levels comparable with HMB. These results could be specifically due to the catabolism of Leu to HMB, since Leu-induced suppression of protein degradation was blocked by the pre-incubation with mesotrione, a specific inhibitor of KICD [40]. Accordingly, these results clearly highlight the importance of KICD as an essential enzyme mediating Leu action and support that Leu’s effects on protein degradation are mediated by its metabolite HMB.

Mitochondria exert important roles in maintaining proper oxygen consumption and ATP production for cellular metabolic activity. Alterations in mitochondrial function and ATP deprivation have been demonstrated to be required to trigger muscle wasting [41, 42]. In particular, dysfunctional mitochondria damage mitochondrial constituents (such as the electron transfer chain) [43] and trigger catabolic signaling pathways that feed-forward to the nucleus to promote the activation of muscle atrophy [44], ultimately leading to cell death. Interestingly, HMB has been proposed as a nutritional supplement to increase muscle health by improving mitochondrial function, since HMB was reported to promote mitochondrial biogenesis of myotubes by about 50% [7, 11, 45]. Furthermore, HMB has been recently reported to be superior to Leu in effectively enhancing mitochondrial function in C2C12 myotubes under anabolic states [12]. Consistently, the current study found that in a starvation model, HMB-treated C2C12 myotubes exhibited significant improvement in mitochondrial function, as manifested by elevated basal respiration, H^+^ leak, ATP production, maximum respiration, and SRC. Moreover, these effects of HMB were more potent than those of Leu or KIC (0.5 mM). Mitochondrial SRC is defined as the difference between basal ATP production and its maximal activity and is considered to be an important aspect of mitochondrial function [46]. In general, high energy-requiring tissues such as skeletal muscle in aging exhibit decreased mitochondrial SRC [47, 48]. A cell with a larger SRC has the ability to generate more ATP and to overcome more stress [49]. Consistent with the current study, previous studies have also demonstrated that mitochondria-targeting therapy by improving mitochondrial respiration is efficient in blocking muscle atrophy [42]. Therefore, we speculated that increased mitochondrial function contributed to the cytoprotective effects of HMB treatment against muscle wasting. Further confirmation comes from the findings that HMB treatment upregulated the protein expression of AMPK and PGC-1α. AMPK-dependent activation of PGC-1α is central for improving mitochondrial function, thus protecting skeletal muscle from atrophy [7, 50, 51]. However, a seemingly paradoxical finding is that AMPK activation induced by higher concentrations of Leu and KIC (10 mM) failed to increase PGC-1α expression. We speculate that this inconsistency may be due to cellular BCAA imbalance induced by higher concentrations of Leu and KIC. It is known that BCAA can affect several synthetic and catabolic cellular signaling cascades resulting in altered phenotypes in mammals [52]. These findings indicate that interplay between BCAA imbalance and PGC-1α activity requires further study. Consistently, high levels of Leu or KIC (10 mM) failed to elicit beneficial effects on mitochondrial oxidative capacity. Since improved mitochondrial function exerts a protective role in muscle health partially via increasing the proportion of slow-twitch fiber types, which are gradually decreased in response to muscle atrophy [53–56], we further assessed the proportion of slow-twitch fibers in muscle cells. As expected, HMB-induced improvement in mitochondrial function was accompanied by increases in the proportion of slow-twitch fibers, as evidenced by upregulated protein expression of MyHC I and downregulated protein expression of MyHC IIb. This novel finding suggests that HMB may suppress muscle protein degradation by elevating the proportion of oxidative fibers and improving mitochondrial function.

To further confirm whether HMB is the active Leu metabolite in regulating mitochondrial function, we assayed the OCR of KICD-transfected C2C12 myotubes in a starvation model. Similar to alterations in protein degradation, over-expressing KICD in C2C12 myotubes potentiated Leu effects on mitochondrial function since in these transfected cells, Leu was approximately as effective as HMB in promoting mitochondrial oxidative capability. These results could be specifically attributed to the conversion of Leu to HMB since Leu failed to elicit any response when myotubes were pre-incubated by mesotrione. These findings are in accordance with the recent literature reporting that Leu’s effects on muscle mitochondrial function were blocked upon KICD knockdown [11]. In addition, alterations in MyHC I mRNA and protein expression as well as immunofluorescence signals showed the similar trends as those of mitochondrial function. These observations indicate that HMB plays beneficial roles in muscle protein degradation partially via improving mitochondrial oxidative capacity.

In summary, our study points out that HMB is more effective than Leu in inhibiting protein degradation and improving mitochondrial oxidative capacity in a starvation model. Over-expressing KICD in C2C12 cells augments Leu response and highlights the catabolism of Leu to HMB in the suppression of protein degradation and in the improvement of mitochondrial function in muscle. These results suggest that in a starvation model, HMB is the active metabolite of Leu and mediates its effects on protein degradation via improving mitochondrial oxidative capacity in muscle cells. These findings may facilitate the development of strategies with HMB supplementation to treat muscle atrophic diseases. Although these intriguing results were obtained in a model cell line system, it is tempting to propose that HMB may serve as an important tool for patients suffering from these atrophic diseases to counteract muscle loss.

## MATERIALS AND METHODS

### Materials

L-Leu (purity ≥ 98.5-101.0%), KIC (purity ≥ 98 %), and HMB free acid (purity ≥ 95%) were purchased from Sigma (St. Louis, MO, USA). TRIzol, DNase I, and SYBR Green detection kit were purchased from Invitrogen (Life Technologies, Carlsbad, CA, USA). Protease inhibitor cocktail was purchased from Roche (Basel, Switzerland). Phosphatase inhibitors were purchased from Thermo Scientific (Waltham, MA, USA). Phosphate Buffered Saline (PBS) and Trypsin were also purchased from Wisent. Mesotrione (2-(4-Mesyl-2-nitrobenzoyl)-1,3-cyclohexanedione)-Pestanal©, catalogue No. 33855) was obtained from Fluka (St. Louis, MO, USA). The growth medium used for cell growth consisted of high glucose Dulbecco’s modified Eagle’s medium (DMEM) purchased from Gibco (Life Technologies, Grand Island, NY, USA), 10% fetal bovine serum (FBS) (Gibco #26050-088), and 1% Antibiotic-Antimycotic (Wisent #450-115-EL). The medium used for differentiation of cells was high glucose DMEM supplemented with 2% horse serum (HS) (Gibco #26050088) and 1% Antibiotic-Antimycotic.

### Cell culture

C2C12 myoblasts were grown in growth medium (which contains 0.8 mM L-Leu) and incubated at 37°C in 5% CO_2_. When the myoblasts reached about 80% confluency, they were differentiated into myotubes by exchanging the growth medium with the differentiation medium. The differentiation medium was changed daily until myotubes were fully formed. After differentiation, myotbues were starved in serum-free medium for 6 h prior to each treatment and the following experiments were performed in this starvation medium.

### Treatment of cells

The dosages of reagents were 0.5 mM or 10 mM for Leu, 0.5 mM or 10 mM for KIC, and 50 μM for HMB. Leu, KIC, and HMB were freshly diluted in medium before treatment of cells. The concentrations of 0.5 mM and 10 mM were selected as treatment concentrations for Leu as they represent physiological and non-physiological concentrations, respectively [22]. Since Leu can be reversibly transaminated to form KIC, the concentrations for KIC were the same as Leu. 50 μM was selected as an appropriate concentration for HMB since about 5~10% KIC can be converted into HMB [23, 24] and since this concentration has been reported to achieve the greatest inhibitory effects on protein degradation [25–27]. After 6 h starvation (a starvation model has been reported in our recent studies [19]), myotubes were then exposed to serum-free media containing indicated agents for 24 h.

### Determination of protein degradation

Protein degradation was measured as previously described with the following modifications [15, 19]. C2C12 cells were plated on 6-well tissue culture plates, differentiated for 6 days, and then starved for 6 h in serum-free medium. Cells were treated with Leu (0.5 mM or 10 mM), KIC (0.5 mM or 10 mM), and HMB (50 μM) and incubated for 24 h. Subsequently, wells were thoroughly washed two times with ice cold PBS, and then incubated for 6 h in buffer A (0.1% bovine serum albumin (BSA), 10 mM HEPES, 2 mM pyruvate, and 5 mM glucose). After 6 h incubation, the medium was collected, and the tyrosine concentration was measured by the HPLC method, the cell monolayer was washed two times with ice cold PBS, and the cells were dissolved in 1 N NaOH. Proteins were measured by the Lowry method using BSA as the standard.

### 3-Methylhistidine level assay

HPLC was used to measure the amount of 3-Methylhistidine (3-MeHis) in media as previously described [28].

### Cell apoptosis assay

The annexin V–fluorescein isothiocyanate (FITC) and PI dual staining technique was used to assess cell apoptosis as previously described [29]. Briefly, the supernatant was removed and 1 mL of 70% cold ethanol was slowly added during vigorous mixing. Samples were stored at 4°C. Cells were washed once with ice-cold PBS and re-suspended in 1 mL of staining reagent containing 50 mg/mL PI and 100 mg/mL RNase for 30 min in the dark. Then, harvested cells were stained PI/Annexin-V-FITC (KeyGEN, Nanjing, China) according to the manufacturer’s instructions and analyzed by flow cytometry (BD FACSCalibur, USA). The degree of apoptosis was quantified as a percentage of the annexin V-positive and PI-negative (annexin V^+^/PI^−^) cells.

### KICD overexpression

To investigate whether HMB is an active Leu metabolite in inhibiting protein degradation, we overexpressed KICD by using the HPD-pEGFP-N1 plasmid, which was obtained from Weier biotechnology co., LTD (Changsha, China). The pEGFP-N1 plasmid was transfected into C2C12 myoblasts along with control vector. After 24 h, the myoblasts were introducted into myotubes with differentiation medium. Then the myotubes were treated with 0.5 mM Leu and 50 μM HMB and incubated for 24 h. The samples were collected for further analysis.

### Quantitative real-time PCR

Total RNA extracted from C2C12 myotubes was reverse-transcribed into cDNA using reverse transcriptase (Takara, Tokyo, Japan) as previously described [30]. The primer sequences used for PCR are designed using the Oligo 6.0 software program. Relative expression of target genes was calculated by the 2^-ΔΔCt^ method [31].

### Western blotting analysis

Total protein extracted from C2C12 myotubes was used to measure the relative protein levels of MAFbx (Proteintech, 12866-1-AP), MuRF1 (Proteintech, 55456-1-AP), MyHC I (Santa Cruz, sc-53089), MyHC IIb (Proteintech, 20140-1-AP), p-AMPKα (Cell signaling technology, 2535s), and PGC-α (Santa Cruz, SC-13067) by the western blotting technique as previously described [30]. Secondary antibodies were purchased from Thermo Scientific Inc (Waltham, MA, USA). The bands of the protein were visualized using a chemiluminescent reagent (Pierce, Rockford, IL, USA) with a ChemiDoc XRS system (Bio-Rad, Philadelphia, PA, USA). The resultant signals were quantified using Alpha Imager 2200 software (Alpha Innotech Corp., San Leandro CA, USA), and the data were normalized with the inner control.

### Oxygen consumption rate measurement

For measurements of mitochondrial respiration, oxygen consumption rates (OCR) was measured as previously described [12]. Briefly, C2C12 myocytes were seeded at density 2.5×10^5^ in 24-well culture plate from SerHorse Bioscience (Billerica, MA, USA), incubated, differentiated, and treated with indicated agents for 24 h as described earlier. After treatment, the cells were washed twice and media was replaced with XF Assay medium from SeaHorse Bioscience containing 4.5 g/L glucose, 1.0 mM sodium pyruvate, and 4.0 mM glutamine (adjusting the pH to 7.35 ± 0.05 using 1 mol/L NaOH). OCR measurements were performed using SeaHorse Bioscience XF Analyzer. All experiments were performed at 37°C. After measurement of basal respiration, oligomycin (1 μM), proton ionophore carbonylcyanide p-trifluoromethoxyphenylhydrazone (FCCP) (1 μM), and rotenone/antimycin A (1 μM) were added sequentially to measure ATP production, maximal respiratory (Max), and nonmitochondrial respiration (NMR), respectively. Thereafter, these respiratory parameters of mitochondrial function were calculated.

### Immunofluorescence and confocal microscopy

After treated with indicated agents for 24 h, C2C12 myotubes were harvested using cold PBS, followed by fixing in 4% paraformaldehyde in PBS at 4 ºC for 10 min. Then, fixed myotubes were attached on slides using of the CytoSpin Cytocentrifuge (Yingtai Ltd, Changsha, China) with spinning down at 1500 rpm for 5 min, followed by drying for 15 min. The slides containing myotubes were washed with PBS for 3×2 min, followed by treatment with 5% (vol/vol) Triton-x100 for 5 min. Then, the slides were washed with PBS for 3×2 min, followed by being incubated with 5% BSA solution to block background staining for 30 min. The slides were incubated in a humidity chamber with MyHC I (sc-53089, Santa Cruz, USA, 1:100) diluted in 5% BSA solution at 4 ºC overnight. The slides were washed 3×2 min with PBS prior to 1 h incubation with 1:1000 dilutions of the CL594-conjugated goat anti-rabbit IgG (H+L) (Proteintech). Nuclei were stained with SlowFade Gold antifade reagent containing 4’,6’-diamidino-2-phenylindole (DAPI) (Sigma, Shanghai, China). Images were taken using confocal electroscope (Zeiss, Germany).

### Statistical analysis

Data obtained from the present study were analyzed by one-way ANOVA using the SAS 8.2 software package, followed by a Duncan’s multiple-range test to determine treatment effects. The results were expressed as mean ± SEM and regarded to achieve statistical significance at *P* < 0.05.
